# Development of predictive score for postoperative dysphagia after emergency abdominal surgery in patients of advanced age

**DOI:** 10.1002/ags3.12716

**Published:** 2023-07-15

**Authors:** Tomohiro Iguchi, Junya Mita, Norifumi Iseda, Shun Sasaki, Noboru Harada, Mizuki Ninomiya, Keishi Sugimachi, Takuya Honboh, Noriaki Sadanaga, Hiroshi Matsuura

**Affiliations:** ^1^ Department of Surgery Saiseikai Fukuoka General Hospital Fukuoka Japan; ^2^ Department of Surgery Oita Red Cross Hospital Oita Japan; ^3^ Department of Surgery and Science, Graduate School of Medical Sciences Kyushu University Fukuoka Japan; ^4^ Department of Surgery Fukuoka City Hospital Fukuoka Japan; ^5^ Department of Hepato‐Biliary Pancreatic Surgery National Hospital Organization Kyushu Cancer Center Fukuoka Japan

**Keywords:** aged, emergency surgery, intramuscular adipose tissue content, postoperative dysphagia, swallowing screening tool

## Abstract

**Aim:**

Postoperative dysphagia after emergency abdominal surgery (EAS) in patients of advanced age has become problematic, and appropriate dysphagia management is needed. This study was performed to identify predictive factors of dysphagia after EAS and to explore the usefulness of swallowing screening tools (SSTs).

**Methods:**

This retrospective study included 267 patients of advanced age who underwent EAS from 2012 to 2022. They were assigned to a dysphagia group and non‐dysphagia group using the Food Intake Level Scale (FILS) (dysphagia was defined as a FILS level of <7 on postoperative day 10). From 2018, original SSTs including a modified water swallowing test were performed by nurses.

**Results:**

The incidence of postoperative dysphagia was 22.8% (61/267). Patients were significantly older in the dysphagia than non‐dysphagia group. The proportions of patients who had poor nutrition, cerebrovascular disorder, Parkinson's disease, dementia, nursing‐care service, high intramuscular adipose tissue content (IMAC), and postoperative ventilator management were much higher in the dysphagia than non‐dysphagia group. Using logistic regression analysis, high IMAC, postoperative ventilator management, cerebrovascular disorder, and dementia were correlated with postoperative dysphagia and were assigned 10, 4, 3, and 3 points, respectively, according to each odds ratio. The optimal cut‐off value was 7 according to a receiver operating characteristics curve. Using 1:1 propensity score matching for high‐risk patients, the incidence of postoperative dysphagia was reduced by SSTs.

**Conclusions:**

The new prediction score obtained from this study can identify older patients at high risk for dysphagia after EAS, and SSTs may improve these patients' short‐term outcomes.

## Abstract 2

This retrospective study included 267 patients of advanced age and was performed to identify predictive factors of dysphagia after emergency abdominal surgery and to explore the usefulness of swallowing screening tools. This study demonstrated that high intramuscular adipose tissue content, postoperative ventilator management, cerebrovascular disorder, and dementia were associated with postoperative dysphagia after emergency abdominal surgery in patients of advanced age. For patients at high risk as identified by our new prediction score that was created based on these risk factors, propensity score‐matching analysis showed that the use of swallowing screening tools can help to prevent postoperative dysphagia.

## 1 INTRODUCTION

Dysphagia is a dysfunction of the digestive system characterized by swallowing impairment.^1^ Dysphagia is commonly seen in patients of advanced age who have experienced long‐term intubation, stroke, or neurodegenerative diseases such as dementia or Parkinson's disease.^2^, ^3^ In addition, dysphagia is a common complication in patients who undergo cervical spine surgery, cardiovascular surgery, or esophagectomy.^4^, ^5^, ^6^ Patients with dysphagia can develop aspiration pneumonia, malnutrition, anorexia, dehydration, and depression.^7^ Moreover, postoperative dysphagia is associated with an increased length of stay and higher 30‐day readmission rate, mortality rate, and healthcare costs.^4^

Sarcopenia is a reported risk factor for swallowing dysfunction. One study showed that preoperative sarcopenia was associated with postoperative swallowing dysfunction as defined by the Food Intake Level Scale (FILS).^6^ Sarcopenic dysphagia is a newly defined pathological condition characterized by the loss of mass and function of swallowing‐related muscle and has recently been attracting attention.^8^ Not only age but also low activity, inflammation, and protein catabolism due to surgical stress and malnutrition may increase the loss of muscle mass, including swallowing‐related muscles. Therefore, it is necessary to carefully evaluate patients for sarcopenia before surgery and determine the most appropriate treatment strategy.

Emergency abdominal surgery (EAS) is carried out to avoid fatal or morbid health consequences of a surgically treatable disease. The morbidity and mortality rates after EAS are usually several‐fold higher than those after elective surgery.^9^ With the aging of the population, the number of older patients who undergo EAS is expected to increase. Postoperative dysphagia seems to be occasionally encountered in patients undergoing EAS, especially those of advanced age. However, in contrast to elective surgery, such patients often have no diagnosis, limited background information, and little time for planning. Hence, surgeons are challenged to improve outcomes after EAS in older patients.

Dysphagia management requires a multidisciplinary approach focusing first on early diagnosis to reduce morbidity, length of stay, and healthcare costs.^10^ Use of swallowing screening tools (SSTs) is the essential first step in identifying the risk of dysphagia and facilitates referral to speech therapists for evaluation and management of dysphagia.^10^ Nurses have recently begun to play a key role in the early identification of patients with dysphagia through the use of SSTs.^11^ However, the importance of SSTs has not yet been fully recognized.

This retrospective study was performed to identify predictive factors of dysphagia after EAS and to explore the usefulness of an SST in older patients at high risk of dysphagia.

## 2 PATIENTS AND METHODS

### 2.1 Patients

**FIGURE 1:**
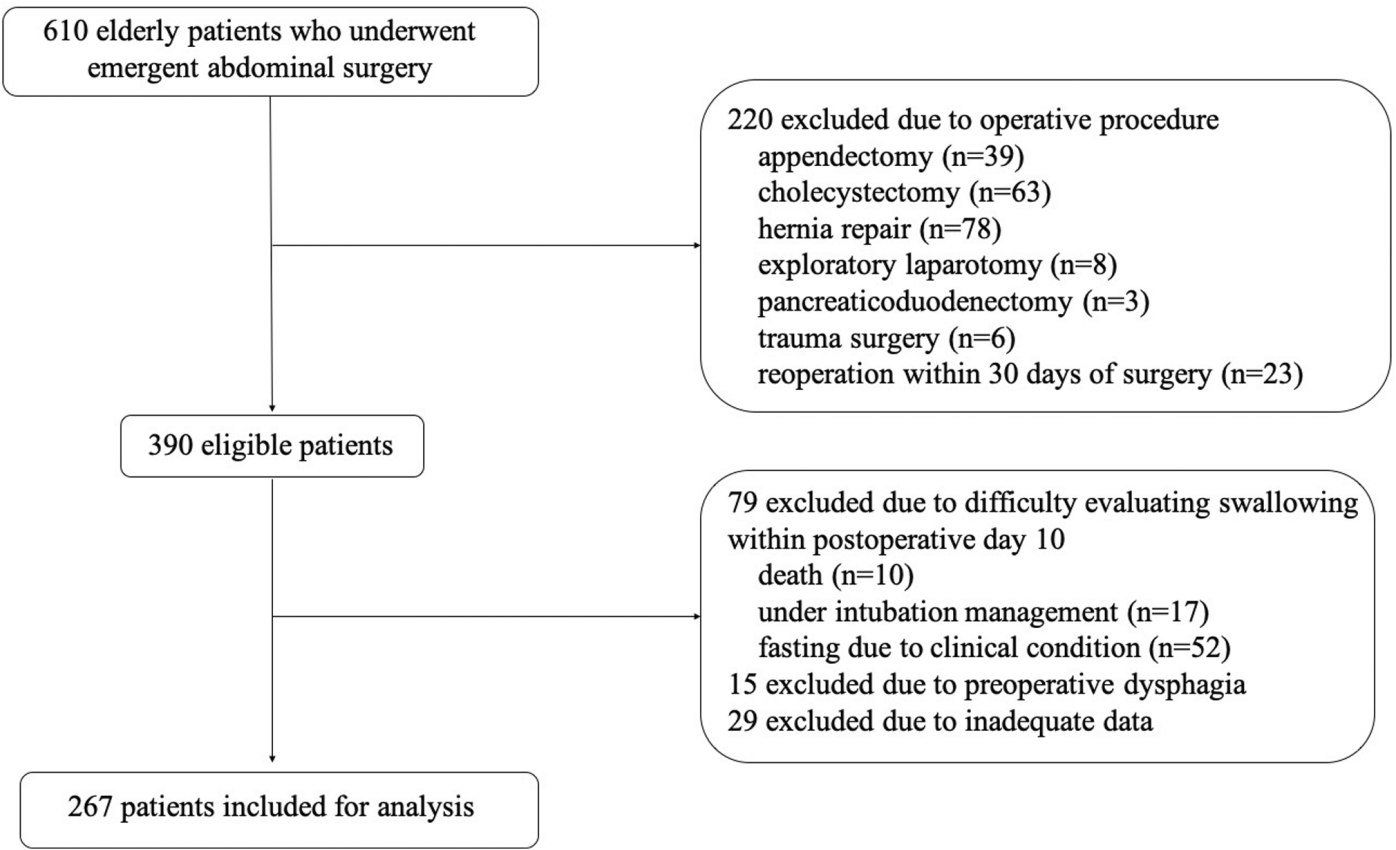
Flow chart of study population. Data collection was performed from October 2012 to September 2022. In total, 267 patients were included in the present study.

We retrospectively enrolled 611 consecutive patients of advanced age (age of ≥75 years) who underwent EAS from October 2012 to September 2022. The exclusion criteria were short‐stay surgery (i.e., appendectomy, cholecystectomy, hernia repair, or exploratory laparotomy), highly invasive surgery (pancreaticoduodenectomy), trauma surgery, reoperation within 30 days of surgery, and difficulty evaluating swallowing function within postoperative day 10 (i.e., disturbance of consciousness, under intubation management, or fasting due to clinical condition). Although preoperative assessment of dysphagia was difficult, patients with clearly impaired swallowing function were excluded. Based on the study enrollment criteria, 267 patients were included in the present study (Figure 1). This study was approved by the Ethics and Indications Committee of Saiseikai Fukuoka General Hospital (2022‐11‐4).

### 2.2 Assessment of swallowing ability

Swallowing ability was assessed using the FILS, which is a 10‐point observer‐rated scale for assessing dysphagia.^6^ We retrospectively collected information from the patients' medical records and obtained their FILS scores on postoperative day 10 or at discharge if discharged within 10 days after surgery. FILS levels 1 to 3 indicate non‐oral intake, levels 4 to 6 indicate oral intake and alternative nutrition, and levels 7 to 10 refer to oral intake alone.^6^ Dysphagia was defined as FILS score of <7 as previously reported.^12^

### 2.3 SST and postoperative swallowing intervention

**FIGURE 2:**
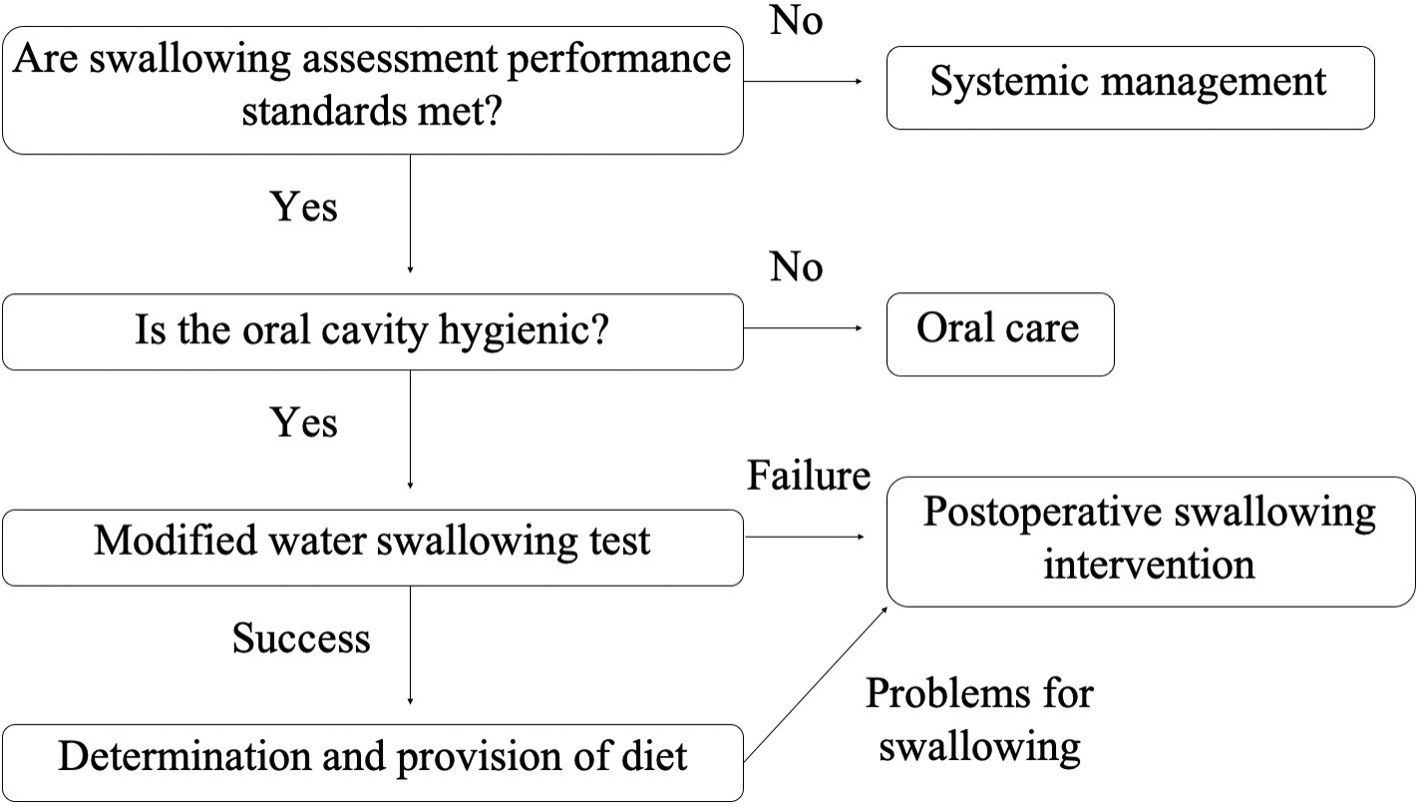
Algorithm for swallowing assessment to identify at‐risk patients. The assessment was performed by nurses certified in dysphagia nursing.

Postoperative swallowing intervention was introduced at the discretion of the primary care physician. From December 2018, an original SST including a modified water swallowing test was performed by a certificated nurse in dysphagia nursing for the purpose of early swallowing intervention and reduction of workload for speech therapists. The algorithm for swallowing assessment to identify patients at risk of dysphagia is shown in Figure 2. If the patient clearly impaired swallowing function or failed the modified water swallowing test, the primary care physician would refer the patient to speech therapists or nurses who would re‐evaluate the patient the next day. As soon as referred, speech therapists evaluated swallowing function by repetitive saliva swallowing test and provided indirect and, if possible, direct training according to the patient's condition. Video endoscopy was also performed in patients with difficulty in evaluating swallowing function by speech therapists, such as those suspected of silent aspiration or vocal cord paralysis and was evaluated using the Hyodo scoring system.^13^ Fifteen of 77 patients who required swallowing intervention were evaluated using video endoscopy.

### 2.4 Measurement of geriatric nutritional risk index and prognostic nutritional index

The geriatric nutritional risk index (GNRI) was calculated as GNRI = 14.89 × albumin (Alb) (g/dL) + 41.7 × [current body weight (kg)/22 × height (m)^2^]. If [current body weight (kg)/22 × height (m)^2^] > 1, the GNRI was set to 1.^14^ The patients' body weight and height were preoperatively obtained from self‐ or family‐declaration. The prognostic nutritional index (PNI) was calculated as PNI = 10 × Alb (g/dL) + 0.005 × total lymphocyte count (per μL).^15^

### 2.5 Imaging analysis

**FIGURE 3:**
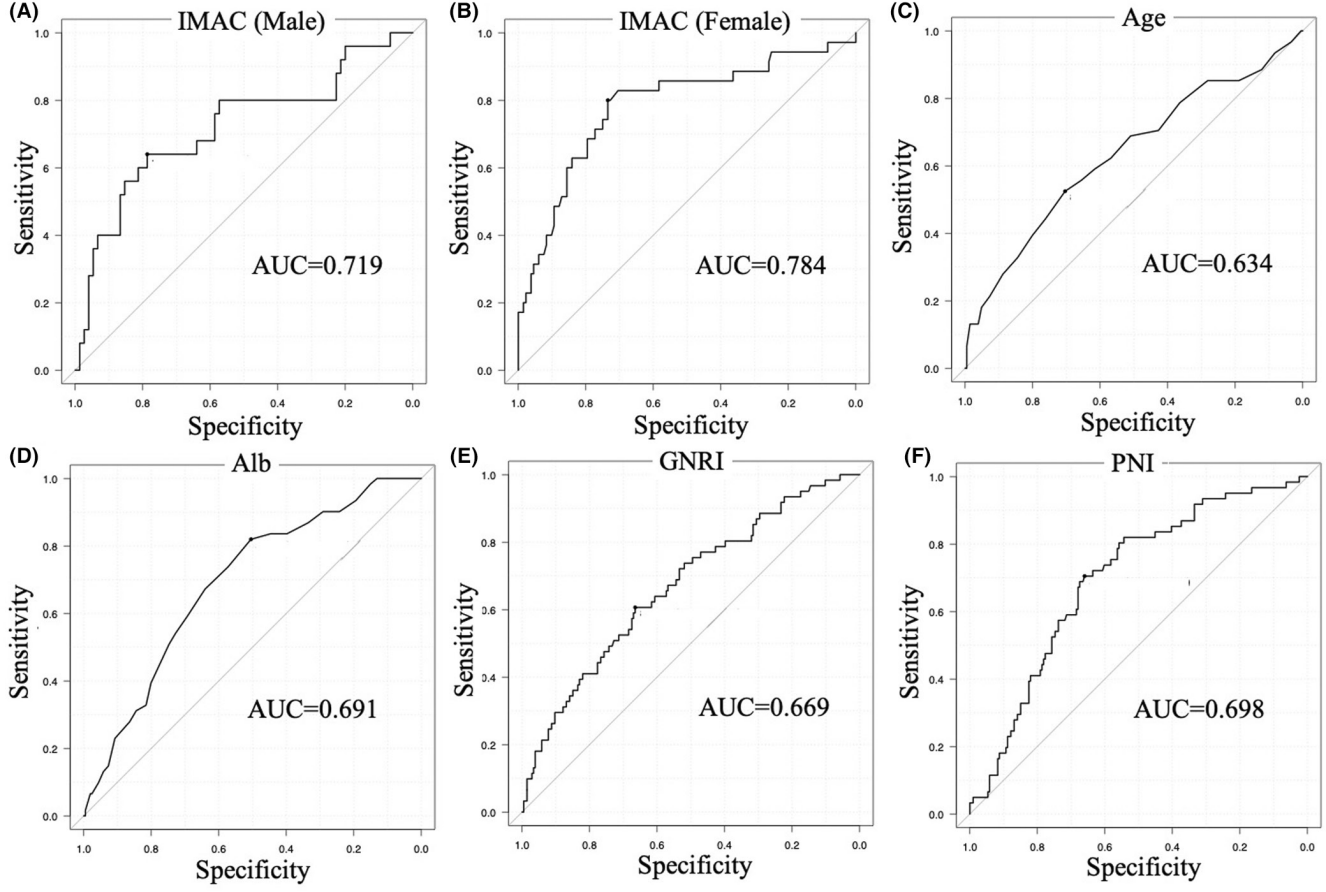
Receiver operating characteristics curves of (A) IMAC for male patients, (B) IMAC for female patients, (C) age, (D) Alb concentration, (E) GNRI, and (F) PNI. Alb, albumin; AUC, area under the curve; GNRI, geriatric nutritional risk index; IMAC, intramuscular adipose tissue content; PNI, prognostic nutritional index.

All computed tomography (CT) imaging before surgery was performed using a multi‐detector CT system (Aquilion PRIME; Canon, Tochigi, Japan). Using a cross‐sectional plain CT scan at the level of the third lumbar vertebra, the quantity and quality of skeletal muscle were evaluated by the psoas muscle index (PMI) and intramuscular adipose tissue content (IMAC), respectively. The PMI was calculated by normalizing the bilateral psoas muscle areas to the squared height of the patient.^16^ Because the correlation between the PMI and postoperative dysphagia was weak, the cut‐off level of the PMI was 6.36 cm^2^/m^2^ for men and 3.92 cm^2^/m^2^ for women according to established criteria for sarcopenia in liver diseases.^17^ It has been reported that this criteria for PMI was related to swallowing function.^6^, ^18^ The IMAC was calculated by dividing the mean CT attenuation value of the bilateral multifidus muscle area (Hounsfield units) by the mean CT attenuation value of four points of subcutaneous fat away from major vessels (Hounsfield units) as previously described.^19^ Increased IMAC implicates a higher amount of adipose deposition in the muscle tissue and thus lower muscle quality. Because of the differences in the IMAC values of male and female patients in this study, the cut‐off point for each sex was set using a receiver operating characteristics (ROC) curve. The cut‐off value for IMAC was −0.321 in men and − 0.273 in women (Figure 3).

### 2.6 Statistical analysis

Continuous and categorical variables were compared with Student's *t* test, the Mann–Whitney *U*‐test, and Fisher's exact test to assess statistical significance (*p* < 0.05). The association between the perioperative factors and the development of postoperative dysphagia was evaluated using logistic regression analysis to adjust confounding factors. The optimal cut‐off values of continuous variables including age, Alb, GNRI, and PNI, were estimated based on an ROC curve analysis (Figure 3). We carried out propensity score matching to adjust for confounding factors between swallowing screening group and non‐swallowing screening group in high‐risk patients for postoperative dysphagia. The propensity score was calculated by logistic regression with the following confounding factors: age; sex; body mass index; history of comorbidities of cancer, cerebrovascular disorder, Parkinson's disease, and dementia; nursing care service; Alb concentration; GNRI; PNI; PMI; IMAC; panperitonitis; operative procedure; and postoperative ventilator management. These groups were then paired 1:1 on these propensity scores using exact matching. The nearest‐neighbor method was used without replacement within a caliper, and the caliper was set to 0.2 of the standard deviation of the logit of the propensity score. We subsequently compared the short‐term outcome. The results were analyzed using EZR on R commander (version 1.60).^20^ A *p* value of <0.05 was considered statistically significant.

## 3 RESULTS

### 3.1 Comparison of clinical factors between patients with and without postoperative dysphagia

Sixty‐one of 261 patients (23.4%) developed postoperative dysphagia. The clinical findings were compared between the dysphagia and non‐dysphagia groups (Table 1). Patients in the dysphagia group were significantly older than those in the non‐dysphagia group (*p* < 0.001). Compared with the non‐dysphagia group, the dysphagia group had a higher percentage of patients with a history of cerebrovascular disorder (*p* = 0.003), dementia (*p* < 0.001), and nursing‐care service (*p* < 0.001); however, there were no significant differences in sex, body mass index, cancer, or Parkinson's disease between the two groups. All nutritional indices including the Alb concentration, GNRI, and PNI were lower in the dysphagia than non‐dysphagia group (all *p* < 0.001). The proportion of patients with a high IMAC (indicating low muscle quality) was much higher in the dysphagia than non‐dysphagia group (*p* < 0.001), whereas there were no significant differences in the PMI (indicating muscle quantity). The median duration of postoperative ventilator management was 4 days (interquartile 2–6 days) in the dysphagia group and 2 days (interquartile 1–3 days) in the non‐dysphagia group. Postoperative ventilator management was needed in the dysphagia group (*p* < 0.001). There were no associations between the prevalence of postoperative dysphagia and panperitonitis or operative procedures. As might be expected in short‐term outcomes, patients in the dysphagia group frequently developed postoperative aspiration pneumonia, required swallowing intervention, and had difficultly leaving the hospital for home (all *p* < 0.001) (Table 1).

**TABLE 1:** Comparison of perioperative factors and postoperative outcomes between postoperative dysphagia group and non‐postoperative dysphagia group.

	Non‐dysphagia group (*n* = 206)	Dysphagia group (*n* = 61)	*p*‐Value
Preoperative factor			
Age, years	83.3 ± 5.3	86.1 ± 6.3	<0.001
Sex, male/female	75/131	25/36	0.549
Body mass index, kg/m^2^	21.2 ± 3.4	20.6 ± 3.4	0.310
Cancer	50 (24.3)	19 (31.1)	0.318
Cerebrovascular disorder	31 (15.0)	20 (32.8)	0.003
Parkinson's disease	2 (1.0)	3 (4.9)	0.080
Dementia	33 (16.0)	27 (44.3)	<0.001
Nursing‐care service	82 (39.8)	40 (65.6)	<0.001
Albumin, g/dL	3.34 ± 0.73	2.87 ± 0.64	<0.001
GNRI	95.18 ± 13.50	86.91 ± 13.02	<0.001
PNI	38.74 ± 8.44	33.33 ± 7.27	<0.001
PMI: male <6.36, female <3.92	180 (87.4)	52 (85.2)	0.668
IMAC: male > − 0.321, female > −0.273	52 (25.2)	45 (73.8)	<0.001
Panperitonitis	50 (24.3)	17 (27.9)	0.615
Operative procedure			
Adhesiolysis	45 (21.8)	5 (8.2)	0.225
Colectomy	48 (23.3)	16 (26.2)	
Ileostomy or colostomy	28 (13.6)	11 (18.0)	
Omental plugging	10 (4.9)	3 (4.9)	
Small bowel resection	57 (27.7)	21 (34.4)	
Other	18 (8.7)	5 (8.2)	
Postoperative ventilator management	38 (18.4)	29 (47.5)	<0.001
Postoperative outcome			
Postoperative complication Clavien–Dindo ≥III	15 (7.3)	8 (13.1)	0.192
Postoperative aspiration pneumonia	3 (1.5)	16 (26.2)	<0.001
Dysphagia rehabilitation intervention	19 (9.2)	58 (95.0)	<0.001
Length of swallowing intervention after extubation	3.95 ± 3.34	3.31 ± 2.56	0.387
Postoperative length of stay	19.6 ± 31.6	25.1 ± 14.1	0.187
Outcome at discharge (transfer/discharge/death)	121/83/2	50/8/3	<0.001

*Note*: Values are presented as number (%) or mean ± SD.

Abbreviations: GNRI, geriatric nutrition risk index; IMAC, intramuscular adipose tissue content; PMI, psoas muscle index; PNI, prognostic nutrition index.

### 3.2 Factors associated with postoperative dysphagia and establishment of a prediction score for postoperative dysphagia

**FIGURE 4:**
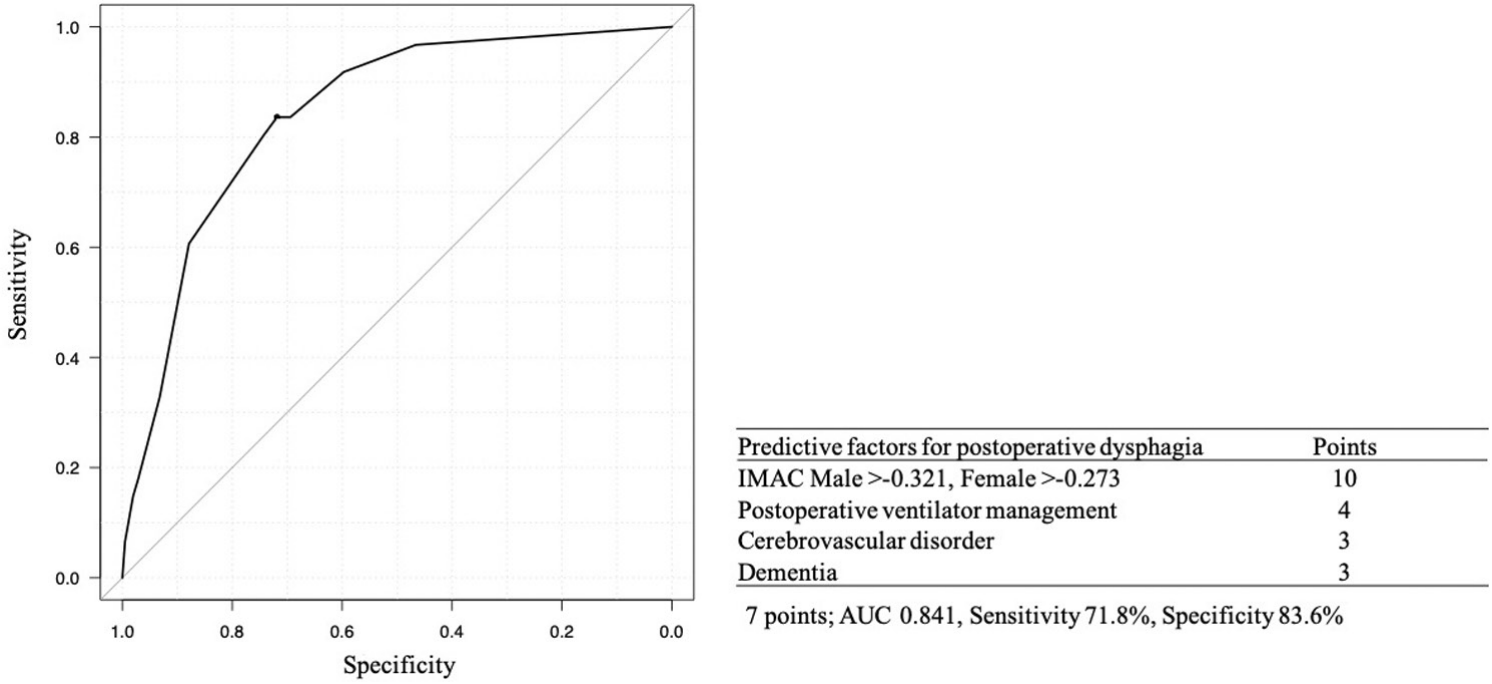
Receiver operating characteristics curves of predictive score for postoperative dysphagia, including high IMAC, postoperative ventilator management, a history of cerebrovascular disorder, and dementia. The optimal cut‐off value was 7 points (AUC, 0.841; sensitivity, 71.8%; specificity, 83.6%). IMAC, intramuscular adipose tissue content; AUC, area under the curve.

In the logistic regression model, high IMAC (odds ratio [OR], 10.1; 95% confidence interval [CI], 4.3–23.8; *p* < 0.001), postoperative ventilator management (OR, 3.9; 95% CI, 1.7–9.2; *p* = 0.002), a history of cerebrovascular disorder (OR, 2.9; 95% CI, 1.1–7.4; *p* = 0.026), and dementia (OR, 2.9; 95% CI, 1.2–7.1; *p* = 0.023) were significantly associated with the overall incidence of postoperative dysphagia (Table 2). To identify patients at high risk for postoperative dysphagia before postoperative management, we established a prediction score for postoperative dysphagia. Based on the estimated coefficients calculated by the logistic regression model, high IMAC, postoperative ventilator management, a history of cerebrovascular disorder, and dementia were assigned 10, 4, 3, and 3 points, respectively. The optimal cut‐off value was determined to be 7 points using ROC curve analysis (area under the curve, 0.841; sensitivity, 71.8%; specificity, 83.6%) (Figure 4).

**TABLE 2:** Multivariate analysis of perioperative risk factors associated with postoperative dysphagia.

Perioperative actors	HR (95% CI)	*p*‐Value
Age (≥87 years)	2.150 (0.928–4.960)	0.0741
Sex (male)	2.27 (0.981–5.270)	0.0555
BMI < 22 kg/m^2^	1.430 (0.545–3.770)	0.4660
Cancer	1.300 (0.492–3.430)	0.5980
Cerebrovascular disorder	2.910 (1.1400–7.420)	0.0258
Parkinson's disease	2.610 (0.306–22.200)	0.3810
Dementia	2.870 (1.160–7.100)	0.0227
Nursing‐care service	0.595 (0.234–1.510)	0.2760
Alb <3.3 g/dL	1.570 (0.440–5.580)	0.4820
GNRI <88.95	1.270 (0.406–3.960)	0.6820
PNI <35.6	2.130 (0.696–6.490)	0.1850
PMI: male <6.36, female <3.92	0.440 (0.139–1.390)	0.1630
IMAC: male >−0.321, female >−0.273	10.100 (4.280–23.800)	0.0000
Panperitonitis	0.696 (0.257–1.880)	0.4750
Operative procedure		
Colectomy	1.630 (0.371–7.160)	0.5180
Ileostomy or colostomy	2.280 (0.473–11.000)	0.3040
Omental plugging	1.070 (0.1080–10.500)	0.9560
Small bowel resection	1.580 (0.407–6.150)	0.5080
Other	1.330 (0.232–7.640)	0.7480
Postoperative ventilator management	3.900 (1.650–9.230)	0.0020

Abbreviations: Alb, albumin; BMI, body mass index; CI, confidence interval; GNRI, geriatric nutrition risk index; HR, hazard ratio; IMAC, intramuscular adipose tissue content; PMI, psoas muscle index; PNI, prognostic nutrition index.

### 3.3 Usefulness of SST for patients at high risk for postoperative dysphagia

Our cohort included 109 patients at high risk for postoperative dysphagia as indicated by a FILS score of ≥7 points. The patients at high risk for postoperative dysphagia were divided into two groups: those in the non‐SST group (*n* = 71) underwent EAS before introduction of the SST (October 2012 to November 2018), and those in the SST group (*n* = 38) underwent EAS after the introduction of the SST (December 2018 to September 2022). Before matching, the percentage of patients with nursing‐care service was higher in the SST than non‐SST group (*p* = 0.001), and patients in the SST group tended to be female and undernourished (Table 3). To reduce the effects of confounding factors in the two groups, propensity score matching analysis was performed for evaluation of short‐term outcomes. Twenty‐four patients in the SST group were matched with 24 patients in the non‐SST group in a 1:1 ratio. After matching, all of their corresponding variables were balanced (Table 3). The short‐term outcomes were then analyzed. Whereas fewer postoperative swallowing interventions were performed and the duration of these interventions was longer in the SST than non‐SST group, a significantly lower proportion of patients developed postoperative dysphagia in the SST than non‐SST group (*p* = 0.036) (Table 3). The incidence of postoperative aspiration pneumonia was lower in the SST than non‐SST group (4.2% vs. 16.7%, respectively), but the difference did not reach statistical significance (Table 3).

**TABLE 3:** Comparisons between swallowing screening group and non‐swallowing screening group among patients at high risk for postoperative dysphagia.

	Before matching		*p*‐Value	Std diff	After matching		*p*‐Value	Std diff
	Non‐SST group (*n* = 71)	SST group (*n* = 38)			Non‐SST group (*n* = 24)	SST group (*n* = 24)		
Preoperative factor								
Age, years	85.3 ± 5.8	85.9 ± 6.1	0.608	0.103	86.4 ± 5.9	85.5 ± 6.7	0.631	0.631
Sex, male/female	29/42	9/29	0.093	0.373	8/16	5/19	0.517	0.284
Body mass index, kg/m^2^	21.5 ± 3.2	21.5 ± 3.5	0.967	0.024	21.8 ± 2.7	21.3 ± 3.7	0.599	0.135
Cancer	17 (23.9)	9 (23.7)	1.000	0.006	7 (29.2)	7 (29.2)	1.000	<0.001
Cerebrovascular disorder	16 (22.5)	13 (34.2)	0.25	0.261	6 (25.0)	5 (20.8)	1.000	0.099
Parkinson's disease	1 (1.4)	3 (7.9)	0.121	0.312	1 (4.2)	0 (0.0)	0.121	0.295
Dementia	28 (39.4)	14 (45.2)	0.839	0.053	12 (50.0)	11 (45.8)	1.000	0.083
Nursing‐care service	40 (56.3)	31 (81.6)	0.011	0.567	18 (75.0)	17 (70.8)	1.000	0.094
Albumin, g/dL	3.34 ± 0.73	2.87 ± 0.64	0.058	0.371	3.12 ± 0.72	2.96 ± 0.74	0.463	0.217
GNRI	92.69 ± 14.46	88.41 ± 14.32	0.084	0.297	92.99 ± 13.80	89.70 ± 15.82	0.343	0.222
PNI	36.02 ± 8.55	34.25 ± 8.87	0.209	0.204	36.28 ± 7.69	35.10 ± 8.86	0.672	0.143
PMI: male <6.36, female <3.92	65 (91.5)	33 (86.8)	0.668	0.152	21 (87.5)	22 (91.7)	1.000	0.137
IMAC: male > − 0/321, female > − 0.273	66 (93.0)	31 (81.6)	0.106	0.346	22 (91.7)	22 (91.7)	1.000	<0.001
Panperitonitis	18 (25.4)	11 (28.9)	0.820	0.081	6 (25.0)	7 (29.2)	0.820	0.094
Operative procedure								
Adhesiolysis	10 (14.1)	5 (13.2)	0.852	0.273	6 (25.0)	3 (12.5)	0.680	0.561
Colectomy	15 (21.1)	10 (26.3)			6 (25.0)	8 (33.3)		
Ileostomy or colostomy	11 (15.5)	5 (13.2)			2 (8.3)	5 (20.8)		
Omental plugging	3 (4.2)	3 (7.9)			1 (4.2)	2 (8.3)		
Small bowel resection	25 (35.2)	10 (26.3)			6 (25.0)	4 (16.7)		
Other	7 (9.9)	5 (13.2)			3 (12.5)	2 (8.3)		
Postoperative ventilator management	27 (38.0)	15 (39.5)	1.000	0.030	8 (33.3)	8 (33.3)	1.000	<0.001
Postoperative outcome								
Postoperative complication Clavien–Dindo ≥III	8 (11.3)	6 (15.8)	0.554		3 (12.5)	3 (12.5)	1.000	
Postoperative dysphagia	38 (53.5)	13 (34.2)	0.070		13 (54.2)	5 (20.8)	0.036	
Postoperative aspiration pneumonia	9 (12.7)	3 (7.9)	0.536		4 (16.7)	1 (4.2)	0.348	
Dysphagia screening intervention		20 (52.6)				10 (41.7)		
Dysphagia rehabilitation intervention	41 (57.7)	16 (42.1)	0.159		14 (58.3)	7 (29.2)	0.080	
Length of swallowing intervention after extubation	3.95 ± 3.34	3.31 ± 2.56	0.239		2.93 ± 1.77	6.00 ± 2.45	0.007	
Postoperative length of stay	19.6 ± 31.6	25.1 ± 14.1	0.187		29.0 ± 40.8	25.2 ± 27.9	0.934	
Outcome at discharge (transfer/discharge/death)	57/14/0	25/10/3	0.034		17/7/0	17/6/1	1.000	

*Note*: Values are presented as number (%) or mean ± SD.

Abbreviations: GNRI, geriatric nutrition risk index; IMAC, intramuscular adipose tissue content; PMI, psoas muscle index; PNI, prognostic nutrition index; SST, swallowing screening tool; Std diff, standardized difference.

## 4 DISCUSSION

This study demonstrated that high IMAC, postoperative ventilator management, cerebrovascular disorder, and dementia were associated with postoperative dysphagia after EAS in patients of advanced age. For patients at high risk as identified by our new prediction score that was created based on these risk factors, propensity score‐matching analysis showed that the use of SST can help to prevent postoperative dysphagia.

IMAC was the most strongly predictive factor of postoperative dysphagia, whereas the PMI was not correlated with postoperative dysphagia in this study. The PMI has been thoroughly studied as a sarcopenic factor. Mayanagi et al.^6^ reported that sarcopenia defined by the PMI was associated with swallowing function in patients undergoing curative resection for esophageal cancer. However, the PMI may not reflect actual muscle mass because myosteatosis (skeletal muscle fat infiltration) increases with aging through leptin signaling, fibro‐adipogenic precursor cells, and mitochondrial dysfunction.^21^ High IMAC reflects both increased intramuscular adipose tissue and loss of muscle mass and is associated with postoperative complications and poor outcomes.^22^, ^23^ Although the biological mechanisms underlying the relationship between IMAC and postoperative dysphagia are not yet known, IMAC is a crucial component of sarcopenia and can be easily measured by CT even in emergency situations.

Postextubation dysphagia is another serious complication.^5^ Mucosal inflammation, direct trauma, muscular atrophy, diminished laryngeal sensory function, and laryngeal injury associated with prolonged intubation (>48 h) can contribute to an increased risk of postextubation dysphagia.^3^ A meta‐analysis of 38 studies showed that the incidence of postextubation dysphagia was 41% regardless of the duration of intubation.^24^ In the present study, postoperative ventilator management, including in patients who were extubated the day after surgery, was significantly associated with dysphagia. It may be necessary to avoid postoperative intubation management as much as possible in advanced‐age patients who undergo EAS.

Swallowing involves regulation of the cranial nerves, central nervous system, and swallow‐related muscles, and dysphagia is a common sequela of stroke. Furthermore, patients commonly develop sarcopenia, anemia, type 2 diabetes mellitus, and osteoporosis after stroke, resulting in poor performance, dementia, and depression.^25^ In this study, there is a possibility that it overlapped with sarcopenia and dementia, and the severity of the stroke sequelae was unknown because of the patients' emergency condition; however, a history of cerebrovascular disorders is a pathological condition that should be noted.

Dementia is associated with functional impairment and disability and has significant physical, psychological, social, and economic impacts.^7^ The prevalence of dysphagia in patients with dementia is variable according to the type (Alzheimer's dementia and vascular dementia) and severity of dementia.^26^ In this study, the type and severity of dementia were not known. It is also necessary to consider the possibility that patients are not aware of problems concerning dementia.

Early diagnosis and intervention of dysphagia can reduce morbidity, the length of stay, and healthcare costs.^10^ For patients undergoing elective surgery, the primary care physician can collect detailed information before surgery, including the patients' organ function, physical function, nutritional status, medical history, and mental and social factors, and treatment decisions based on this information may improve the patients' outcome. In the emergency setting, however, patients often have limited background information, including information regarding their swallowing function. Furthermore, patients of advanced age may have some degree of dysphagia and silent aspiration, although they are often unaware of the problem.^27^ Several studies showed that preoperative sarcopenia was associated with postoperative dysphagia in patients undergoing elective surgery^5^, ^6^; to the best of our knowledge, however, no reports to date have predicted the prevalence of dysphagia after EAS. A new scoring system that can be easily evaluated from limited information even in emergency situations would be useful for obtaining informed consent, preoperative discussion, decision‐making, and postoperative planning for such patients. For patients at high risk for postoperative dysphagia, less invasive alternative treatment, early diagnosis and intervention of dysphagia, and postoperative nutritional support may improve the patients' outcome.

Various SSTs such as the 10‐Item Eating Assessment Tool and the modified water swallowing test have shown high sensitivity and specificity in the assessment of patients with dysphagia.^28^, ^29^ An appropriately sensitive screening will benefit healthcare by reducing the patients' costs and the burden on speech therapists with unnecessary referrals. Therefore, we introduced a nurse‐led original SST including a modified water swallowing test. Introduction of the SST resulted in fewer postoperative swallowing interventions and significantly longer time until intervention by a speech therapist, nevertheless, the results suggested that postoperative dysphagia can be prevented by using the SST after matching. Three possible reasons for this are as follows. First, appropriate referrals of intervention increased due to the raising awareness of the primary care physician in swallowing disorders and workload for speech therapists. Second, nurses repeatedly screened patients' eligibility for intervention by speech therapists, which may have contributed to the increased time to intervention and led to swallowing training. Third, introduction of the SST raised nurses' awareness in swallowing disorders, helping to prevent dysphagia.^30^

The present study has two main limitations. First, this was a retrospective single‐center study, and the small number of patients with limited information resulted in insufficient power to conclusively determine the predictive factors for dysphagia after EAS. Second, SSTs were not used for all patients at high risk for postoperative dysphagia. A well‐planned prospective multicenter study is needed to determine the efficacy of SSTs for preventing dysphagia in patients who undergo EAS.

In conclusion, the new prediction score obtained from this retrospective study can help to identify older patients at high risk for dysphagia after EAS and be useful for obtaining informed consent, preoperative discussion, decision‐making, and postoperative planning, and SSTs may improve the short‐term outcome in these patients.

## AUTHOR CONTRIBUTIONS

Tomohiro Iguchi: study concept and design, drafting of manuscript; Junya Mita: study concept and design; Norifumi Iseda: data collection and critical revision of the manuscript; Shun Sasaki: statistical analysis; Noboru Harada: data collection; Mizuki Ninomiya: data collection; Keishi Sugimachi: critical revision of the manuscript; Takuya Honboh: data collection; Noriaki Sadanaga: critical revision of the manuscript; Hiroshi Matsuura: final approval of the manuscript.

## FUNDING INFORMATION

The work was not supported by any grant or foundation.

## ETHICS STATEMENTS

Approval of the research protocol by an Institutional Reviewer Board: The protocol for this retrospective research project was approved by a suitably constituted ethics committee of the institution (Committee of Saiseikai Fukuoka General Hospital, approval no. 2022‐11‐4), and it conforms to the provisions of the Declaration of Helsinki.

Informed Consent: Patients were not required to give informed consent for inclusion in the study because the analysis used anonymous clinical data that were obtained after each patient agreed to treatment by written consent.

Registry and the Registration No. of the study/trial: Not applicable.

Animal Studies: Not applicable.

## CONFLICT OF INTEREST STATEMENT

The authors have no conflicts of interest to declare.
